# MicroRNA-92b targets tumor suppressor gene FBXW7 in glioblastoma

**DOI:** 10.3389/fonc.2023.1249649

**Published:** 2023-09-11

**Authors:** Nilmary Grafals-Ruiz, Annelis O. Sánchez-Álvarez, Yasmarie Santana-Rivera, Eunice L. Lozada-Delgado, Robert J. Rabelo-Fernandez, Christian I. Rios-Vicil, Fatima Valiyeva, Pablo E. Vivas-Mejia

**Affiliations:** ^1^ University of Puerto Rico Comprehensive Cancer Center, San Juan, Puerto Rico; ^2^ Department of Biochemistry, University of Puerto Rico, San Juan, Puerto Rico; ^3^ Department of Physiology, University of Puerto Rico, San Juan, Puerto Rico; ^4^ Dentistry School, University of Puerto Rico, San Juan, Puerto Rico; ^5^ Departments of Biology, University of Puerto Rico, San Juan, Puerto Rico; ^6^ Department of Neurosurgery, University of Puerto Rico, San Juan, Puerto Rico

**Keywords:** glioblastoma, microRNA-92b, FBXW7, tumor suppressor, cancer

## Abstract

**Introduction:**

Glioblastoma (GBM) is a highly aggressive and lethal primary brain tumor. Despite limited treatment options, the overall survival of GBM patients has shown minimal improvement over the past two decades. Factors such as delayed cancer diagnosis, tumor heterogeneity, cancer stem cell survival, infiltrative nature of GBM cells, metabolic reprogramming, and development of therapy resistance contribute to treatment failure. To address these challenges, multitargeted therapies are urgently needed for improved GBM treatment outcomes. MicroRNAs (miRNAs) are small non-coding RNAs that regulate gene expression. Dysregulated miRNAs have been identified in GBM, playing roles in tumor initiation, progression, and maintenance. Among these miRNAs, miR-92b (miRNA-92b-3p) has been found to be overexpressed in various cancers, including GBM. However, the specific target genes of miR-92b and its therapeutic potential in GBM remain poorly explored.

**Methods:**

Samples encompassed T98G, U87, and A172 human GBM cell lines, GBM tumors from Puerto Rican patients, and murine tumors. In-situ hybridization (ISH) assessed miR-92b expression in patient tumors. Transient and stable transfections modified miR-92b levels in GBM cell lines. Real-time PCR gauged gene expressions. Caspase 3 and Trypan Blue assays evaluated apoptosis and viability. Bioinformatics tools (TargetScanHuman 8.0, miRDB, Diana tools, miRWalk) predicted targets. Luciferase assays and Western Blots validated miRNA-target interactions. A subcutaneous GBM Xenograft mouse model received intraperitoneal NC-OMIs or miR92b-OMIs encapsulated in liposomes, three-times per week for two weeks. Analysis utilized GraphPad Prism 8; statistical significance was assessed using 2-tailed, unpaired Student’s t-test and two-way ANOVA as required.

**Results:**

This study investigated the expression of miR-92b in GBM tumors compared to normal brain tissue samples, revealing a significant upregulation. Inhibition of miR-92b using oligonucleotide microRNA inhibitors (OMIs) suppressed GBM cell growth, migration, and induced apoptosis, while ectopic expression of miR-92b yielded opposite effects. Systemic administration of liposomal-miR92b-OMIs in GBM xenograft mice resulted in reductions in tumor volume and weight. Subsequent experiments identified F-Box and WD Repeat Domain Containing 7 (FBXW7) as a direct target gene of miR-92b in GBM cells.

**Discussion:**

FBXW7 acts as a tumor suppressor gene in various cancer types, and analysis of patient data demonstrated that GBM patients with higher FBXW7 mRNA levels had significantly better overall survival compared to those with lower levels. Taken together, our findings suggest that the dysregulated expression of miR-92b in GBM contributes to tumor progression by targeting FBXW7. These results highlight the potential of miR-92b as a therapeutic target for GBM. Further exploration and development of miR-92b-targeted therapies may offer a novel approach to improve treatment outcomes in GBM patients.

## Introduction

Glioblastoma (GBM) is the second most common primary brain tumor (next to benign meningiomas) and the most common of all malignant primary brain tumors (1). GBM accounts for 54% of all diagnosed gliomas and 16% of all primary brain tumors in adults (1). GBM is also the deadliest of all malignant primary brain tumors, responsible for over 10,000 annual deaths in the US alone (2). Patients diagnosed with GBM live only 3-6 months if left untreated and up to two years with standard-of-care therapy (2). The current standard of care for GBM patients consists of maximal tumor resection in combination with radiotherapy (XRT) and temozolomide (TMZ) chemotherapy. Radiosurgery and TMZ combination are also possible (3). Nevertheless, about 90% of GBM patients acquire resistance to TMZ due to the overactivation of the O^6^-methylguanine-DNA methyltransferase (MGMT) (4). Due to the TMZ chemoresistance, GBM patients do not respond to the second round of TMZ treatment.

Many other therapeutic approaches are available in the clinic or under investigation. Some of them include Bevacizumab with concomitant Irinotecan, Gliadel (carmustine) or implanted wafer disk therapy, localized administration of chemotherapies via implants or experimental catheter-based infusions (convection-enhanced delivery, CED), and the use of tumor treating fields (TTF) (5–9). These treatments are sometimes combined with TMZ. Unfortunately, the overall survival of GBM patients has barely improved over the last 20 years (10, 11). Therefore, novel, and more effective therapies are urgently needed.

Over the last decade, miRNAs have gained significant popularity as potential targets against cancer and other diseases. MiRNAs are endogenous small non-coding RNAs (22 nucleotides in length) that regulate gene expression at the post-transcriptional level (12, 13). Evidence indicates that miRNAs regulate more than 50% of protein-coding genes (14, 15). As miRNAs bind to mRNAs (mostly in the 3’ UTR) by partial complementarity, a single miRNA can potentially regulate the expression of multiple genes simultaneously (16). Cumulating evidence indicate that deregulated miRNAs and their target genes contribute to GBMs’ initiation, progression, and infiltration ability (4, 17–19). Many of these miRNAs have been proposed as targets for GBM therapy.

An early report of our research team identified miR-92b as an upregulated miRNA in human GBM tumor samples compared to lower-grade gliomas and healthy brain tissues (20). MiR-92b has also been implicated in neuroblastoma tumorigenesis and in the regulation of critical tumor-suppressive genes altered in GBM (21, 22). However, the genes through which miR-92b exerts its biological effects in GBM cells have not been fully elucidated. Here, we performed *in vitro* assays, in silico studies, western blots and luciferase reporter assays and found that miR-92b regulates the expression of FBXW7, a recognized tumor suppressor gene. Additionally, we showed that targeting miR-92b with a liposomal-OMI formulation reduced tumor growth in a subcutaneous GBM mouse model.

## Materials and methods

### Chemicals and reagents

Dulbecco’s Phosphate Buffer Saline (PBS) and Tert-butanol were purchased from Sigma-Aldrich (St. Louis, MO). DSPE-PEG-2000 (1,2-distearoyl-sn-glycerol-3-phosphoethanolamine-N-[methoxy(polyethylene glycol)-2000]), DOPC (1,2-dioleoyl-sn-glycero-3-phosphocholine), and cholesterol were purchased at Avanti Polar Lipids (Alabaster, AL). MirVana oligonucleotide miRNA inhibitors (OMIs) were purchased from Thermo Fisher (Waltham, MA), which includes a Negative Control #1 OMIs (NC-OMIs) and the microRNA-92b-3p oligonucleotide inhibitor (miR92b-OMIs).

### MiRNA-92b detection in GBM patients through *in situ* hybridization

We used the *in-situ* hybridization (ISH) technique to evaluate and localize the expression of miR-92b in GBM tumors from Puerto Rican patients. This technique was performed by using the MiRCURY LNA miRNA Detection Probes Kit (Qiagen, MD, USA) following the manufacturer’s specifications. In brief, 10 µm thick brain slices (previously paraffin-embedded) were deparaffinized in xylene and rehydrated in ethanol (100%, 90%, 80%, and 70%). Tissue sections were pre-digested with proteinase-K at 37°C for 10 minutes and hybridized with 10 nM of miRNA LNA Detection Probes in miRNA ISH buffer (provided by the kit) at 60°C for 60 minutes. This miRNA LNA Detection Probes targeted the miR-92b-3p sequence (Cat# YD00610819, MIMAT0003218: 5’-UAUUGCACUCGUCCCGGCCUCC) or a scramble sequence (Cat# YD00699003) as control. After hybridization, tissues were washed in 5X saline-sodium citrate (SSC) buffer at ~25°C (room temperature, RT) and blocked with 2% Sheep Serum (in PBS-T) for 30 minutes RT. Then, they were incubated with sheep anti-DIG-AP (1:500 in 2% Sheep Serum) for 60 minutes RT. Next, samples were incubated with AP substrates (Levamisol 200 µM in MiliQ-Water and NBT-BCIP tablets) for 2 hours at 30°C in a humid chamber, and the reaction stopped with KBT buffer (2 times for 5 minutes each). Finally, tissues were counterstained with Nuclear Fast Nuclear Counterstain (cat# H-3403, Vector laboratories, CA, USA), dehydrated in ethanol solutions (70%, 80%, 90%, and 100%), and mounted with Permount Mounting Media (Cat#SP15, Fisher Chemicals, NH, USA). Pictures of the tissues were taken with a Nikon DS-Qi2 Camera under a Nikon Eclipse E400 microscope at 20X magnification. Images were analyzed with the NIS-Element Microscope Imaging Software by comparing the amount of MiR-92b positive cells in GBM brain tissues vs. normal brain tissues.

### Cell lines and culture conditions

The T98G, U87 (U87 MG or HTB-14), and A172 human GBM cell lines were purchased from the American Type Culture Collection (ATCC, VA). Cell lines were grown as adherent cells and maintained in DME/F12 Media from HyClone Lab (Logan, UT) supplemented with 10% of fetal bovine serum (FBS) (Thermo Fisher) and 1% of penicillin/streptomycin (Thermo Fisher) at 37°C in a humid atmosphere with 5% CO_2_ (standard culture conditions). Cells used for *in vitro* experiments had confluences of 75-85%.

### Transient and stable transfections

For transient transfection, T98G (3.5 x 10^4^ cells/mL) or U87 cells (5.0 x 10^4^) were seeded, and the next day, oligonucleotide microRNA inhibitors (OMIs) targeting miR-92b (miR92b-OMIs) or negative control OMIs (NC-OMIs) were mixed with Lipofectamine RNA iMAX (Thermo Fisher) at a 1:1 ratio (v/v), and Opti-MEM media (Thermo Fisher) were added to cells. After incubating cells for seven hours, Opti-MEM was replaced with DMEF/12 media supplemented with 10% FBS. Experiments with transfected cells were performed 24 hours, 48 hours, or 72 hours after transient transfection. MiR-92b downregulation in cells was monitored with Taq Man quantitative PCR (qPCR).

For stable transfections, A172 cells (3.5 x 10^4^ cells/mL) were seeded in 6-well plates and maintained at standard culture conditions for 24 hours. The next day, Opti-MEM media was added with a mixture of 3 µg of MiR92b-Vector (pEZX-MR03-miR92b) or Empty-Vector (pEZX-MR03) from Genecopoeia (Rockville, MD) and Lipofectamine RNAiMAx at a 1:1 ratio (v/v). After 7 hours, the Opit-MEM was replaced with DMEF/12 supplemented with 10% FBS. Since these vectors contain a puromycin resistance cassette, cell clones were selected and maintained with DMEF/12 media supplemented with Puromycin (10 µg/mL). These vectors also contain a green fluorescent protein (GFP) gene co-expressed with the target miRNA. Thus, transfection was confirmed by localizing GFP-positive cells under a fluorescence microscope and Taq Man qPCR to evaluate miR-92b overexpression.

### RNA isolation, cDNA synthesis, and real-time PCR

Total RNA (including miRNAs) was isolated with the mirVana miRNA Isolation Kit (Thermo Fisher) per the manufacturer’s instructions. Ten nanograms (ng) of RNA were used for cDNA synthesis with the TaqMan MicroRNA Reverse Transcription Kit (Thermo Fisher) in an Applied Biosystems Veriti 96 well Thermal Cycler (16°C for 30 minutes, 42°C for 30 minutes, 85°C for 5 minutes, and 4°C for 15 minutes). One µL of cDNA was added to TaqMan Universal Master Mix II, with UNGs (Thermo Fisher) and primers for miR-92b or U48 (internal control) (Lozada-Delgado et al., 2018; Rivera-Díaz et al., 2015b). PCR was performed on Step One Plus Real-Time PCR System (Thermo Fisher), and data were processed with the StepOne V2.3 Analysis Software. The relative expression of miR-92b was calculated by the ΔΔCt method (23) using the U48 samples as the internal control (24).

### Cell growth and proliferation studies

The effect of miR-92b inhibition on GBM cell viability was assessed with the CyQuant Proliferation Assay Kit (Thermo Fisher). U87 cells were seeded on clear 96-well plates (5 x 10^4^ cells/mL) from Eppendorf (Hamburg, Germany). The next day, cells were transfected with different concentrations of NC-OMIs or miR92b-OMIs (3 nM, 6 nM, 12 nM, 25 nM, 50 nM, 100 nM, and 200 nM). Seventy-two hours post-transfection, CyQUANT GR cell dye was added to cells and incubated for one hour at standard cell conditions, according to the manufacturer’s instructions. Afterward, the number of cells was analyzed by measuring fluorescence at 485/530 nm (Excitation/Emission) with the Varioskan Flash Spectral Scan Multimode Reader (Thermo Fisher). The number of cells was determined using a standard curve with a known number of cells.

To determine the effect of miR-92b-OMIs in the proliferation of GBM cells at different time points, U87 cells (5.0x10^4^ cells/mL) were seeded on 6-well plates and maintained under standard cell growth conditions. The following day cells were transfected with 100 nM of NC-OMIs and miR92b-OMIs as previously specified. After transfection, cell concentration (cells/mL) was calculated at 24, 48, and 72 hours (25).

The effect that miR-92b has on the ability of a single GBM cell to grow into a colony was assessed by clonogenic assays in T98G (after transfection with NC-OMIs or miR92b-OMIs) and A172 cells (after transfection with miR92b-Vectors or Empty-Vectors). First, T98G transfected cells or A172 clones were detached with trypsin (0.25%) and counted to seed 1,000 cells in 10-cm Petri dishes. Next, cells were maintained in standard culture conditions for ten days and stained with 0.5-1.0% crystal violet (in methanol). Then, five random areas (21 mm diameter circles) were chosen for analysis using an Eclipse TS100 microscope from Nikon (Minato, Tokyo, Japan) (24). Colonies of at least 50 cells were counted.

### Cell migration studies

A total of 5.5x10^4^ cells/mL of U87 cells were seeded onto a 6-well plate, and the following day, they were transfected with 50 nM of either NC-OMIs or miR-92b-OMIs. After 24 hours post-transfection, a sterile pipette tip of 1000 µL was used to create a scratch in the middle of each well containing cells. Subsequently, photographs were captured using a Nikon Eclipse TS100 microscope, and the percentage of wound closure was calculated at 12 and 24 hours.

### Trypan blue viability and caspase-3 activity assays

U87 cells were seeded onto 10 cm Petri dishes at a density of 5.5 X 10^4^ cells/mL and were maintained under standard cell culture conditions. The following day, the cells were transfected with 100 nM (final concentration) of either NC-OMIs or miR-92b-OMIs, as previously described. Cells were collected at 0-hr, 24-hr, and 48-hr after OMIs transfection. Trypan blue (0.4% solution in PBS) was added to the cell suspension to count (under microscope) the number of viable cells in each condition. Another group of cells were collected 48 hours after OMIs transfection to measure the caspase-3 levels using the fluorometric Caspase-3 Assay Kit (cat# ab39383, Abcam Waltham, MA, USA) according to the manufacturer’s instructions. Briefly, 100 µg of protein lysates were added to each well of a black 96-well plate, followed by the addition of 50 µL of Reagent Buffer (with 10 mM DTT) to each sample. The samples were then incubated with 5 µL of DEVD-AFC (AFC: 7-amino-4-trifluoromethyl coumarin) substrate (50 µM) for 2 hours at 37°C, after which caspase 3 activity was determined by measuring fluorescence at 505 nm.

### MiR-92b target prediction and validation

The binding of miR-92b to potential messenger RNAs (mRNAs) was assessed using five different miRNA target prediction softwares (TargetScanHuman 8.0, miRDB, Diana tools, and miRWalk), and the miRTarbase database. The top 250 genes predicted in each program were selected, and genes identified in more than three of the prediction software were chosen and validated through qPCR analysis. For this last step, a 384-well PrimePCR plate from (Bio-Rad) was customized and included specific primers for each gene, and PCR controls. Total RNA from U87 cells transfected with NC-OMIs or miR92b-OMIs was reversed transcribed with the iScript cDNA Synthesis Kit (Bio-Rad). Gene expression was detected using the iTaq Universal SYBR Green Supermix Kit and the CFX384 Touch Real-Time PCR Detection System (Bio-Rad) according to the manufacturer’s specification (denaturation at 95°C for 2 minutes, annealing/extension and plate read at 60°C for 5 seconds x 40 cycles, and melting curve analysis at 60°C for 30 seconds). QPCR data were collected and analyzed using the CFX Maestro Software (Bio-Rad). Experiments were performed in triplicates. The relative expression of the mRNA targets was quantified by the ΔΔCt method using β-Actin as the internal control.

### Western blots

U87 cells were seeded (5 x 10^4^ cells/mL) on 10-cm Petri dishes. Twenty-four hours later, cells were transfected with NC-OMIs or miR92b-OMIs and collected 48 hours post-transfection. Cells were lysed by incubating them on ice for 30 minutes in a cold lysis buffer (1% Triton X, 0.4 mM NaVO_4_, 0.4 mM NaF, and Sigma-Aldrich’s protease inhibitor cocktail) with periodical vortex mixing. The proteins were isolated after recovering the supernatant from centrifugation (13,000 rpm for 15 minutes at 4°C). Then, protein concentration was quantified with the Bio-Rad DC Protein Assay Kit according to the manufacturer’s specifications. Fifty micrograms of protein per sample (previously resuspended in lysis buffer and loading dye) were served on 12% SDS-PAGE gels, separated according to their molecular weights, and blotted on nitrocellulose membranes, where they were blocked with 5% non-fat milk for 1 hour at room temperature, and probed with rabbit polyclonal anti-FBXW7 (1 µg/mL) from Novus Biologicals (Centennial, CO), ASB5 from R&D Systems (Minneapolis, MN), ZNF776 from Abnova (Taipei, TW), TEF from Sigma Aldrich (St. Louis, MO), Bcl-2, PARP, PTEN and c-MYC antibodies were from Cell Signaling Technology (Danvers, MA) overnight at 4°C. Membranes were washed three times for 5 minutes each and incubated in anti-rabbit IgG horseradish peroxidase (HRP) from Cell Signaling Technology (Danvers, MA) at a concentration of 1:5,000 in bovine serum albumin (BSA) during 1 hour at room temperature. Then, enhanced chemiluminescence substrates were added to bound antibodies and blots autoradiographed with a FluorChemTM 8900 from Alpha Innotech Corporation (San Leandro, CA).

### Luciferase assay

We used a dual-Luciferase Reporter Assay System Kit (Promega, Madison, WI) as per the manufacturer’s instructions. Briefly, U87 cells were seeded on 6-well plates at a concentration of 7.5X10^4^ cells/mL and maintained under standard cell culture conditions for 24 hours. For luciferase assay, 50 nM of NC-OMIs or miR92b-OMIs were transfected using Lipofectamine RNAi Max at a 1:1 volume-to-volume ratio (OMIs : LipofectamineRNA iMAX) in OPTIMEM media and incubated for eight hours. Subsequently, the cells were transfected with 0.05 µg of Firefly/Renilla luciferase reporter mammalian expression and 1.5 µg of vector from Origene (SC215694) with Lipofectamine (1:2.5 ratio 3’UTR vector: Lipofectamine) in OPTIMEM. This vector contains the human 3’ UTR of FBXW7 (NM_033632). Twenty-four hours later, media was replaced with DMEF/12 supplemented media, and cells were allowed to recover under standard cell culture conditions for 48 hours. Subsequently, the firefly/renilla activity was measured using the Glomax 20/20 luminometer.

### Subcutaneous GBM Xenograft mouse model and treatment

The animals used in this experiment were handled according to the Institutional Animal Care and Use Committee (IACUC) of the University of Puerto Rico, Medical Sciences Campus (protocol number: A870110). We established a subcutaneous GBM xenograft mouse model by injecting U87 cells (2 x 10^6^ cells/200 µL of PBS and Matrigel Mixture) into the right dorsal flank of 6-week-old male (n=7) and female (n=5) BALB/c nude mice from Taconic Biosciences (Rensselaer, NY). A week after tumor implantation, mice were treated with intraperitoneal (i.p.) injections containing 2.5 µg of NC-OMIs (n=5) or miR92b-OMIs (n=7), encapsulated in DOPC-Cholesterol-PEG-based liposomes (24, 26). Treatments were administered three times a week for seventeen days. Along with treatment, the length (L), the width (W), and the height (H) of each subcutaneous tumor were measured with a caliper; tumor volumes of each mouse were calculated with the ellipsoid volume formula [(*L x W x H*) (π / 6)]. Mice were euthanized four weeks after tumor implantation, and tumors were weighed and harvested.

### Interrogation of the patient databases

The Cancer Genome Atlas (TCGA) database (https://www.cancer.gov/ccg/research/genome-sequencing/tcga) was used to interrogate the miR-92b levels and to construct Kaplan-Meier patient survival graphs. Level 3 microRNA expression data from 479 glioblastomas were obtained from TCGA (platform code: H-miRNA_8 × 15K; Agilent 8 x 15K Human miRNA-specific microarray). Statistical analyses were performed in R (version 3.0.1; http://www.r-project.org/), and the statistical significance was defined as a P value of <0.05. The log-rank test was employed to determine the relationship between miR-92b expression and overall survival (OS). The entire population (163 samples) was randomly split into training (2/3) and validation (1/3) cohorts. In each cohort, patients were divided into percentiles according to miR-92b expression. Then using the training set, any cut-off (0.38) between percentiles of 25th and 75th were considered as statistically significant. The statistical significance was corroborated with the validation set using the same cut-off. The expression of FBXW7 in GBM patients was interrogated using the Gene Expression Profiling Interactive Analysis (GEPIA) database (http://gepia.cancer-pku/cn/) developed by Zefang Tang, Chenwei Li and Boxi Kang of Zhang Laboratory at Peking University. The GEPIA2 database includes the RNA sequencing expression data of 9,736 tumors and 8,787 normal samples from “The Cancer Genome Atlas” (TCGA), and the “Genotype-Tissue Expression” (*GTEx*) databases. GEPIA2 provides information of tumor *vs*. normal differential expression and patient survival analysis. p-values < 0.05 were considered statistically significant. We also assessed the FBXW7 expression using the internet searchable data visualization tool, GlioVis (http://gliovis.bioinfo.cnio.es/). GlioVis include various brain tumors expression datasets, including the TCGA, the Chinese Glioma Genome Atlas (CGGA), and Rembrandt (27).

### Statistical analysis

All experiments were performed in triplicates and analyzed using GraphPad Prism 8 (GraphPad Software, La Jolla, CA, USA). Statistical differences were determined using a 2-tailed, unpaired Student’s t-test and two-way ANOVA tests were performed as per the requirement of the analysis * p ≤ 0.05, ** p ≤ 0.01, *** p ≤ 0.001, **** p ≤ 0.0001. p-value of less than 0.05 was considered statistically significant.

## Results

### MiR-92b expression in GBM patients and GBM cell lines

**Figure 1:**
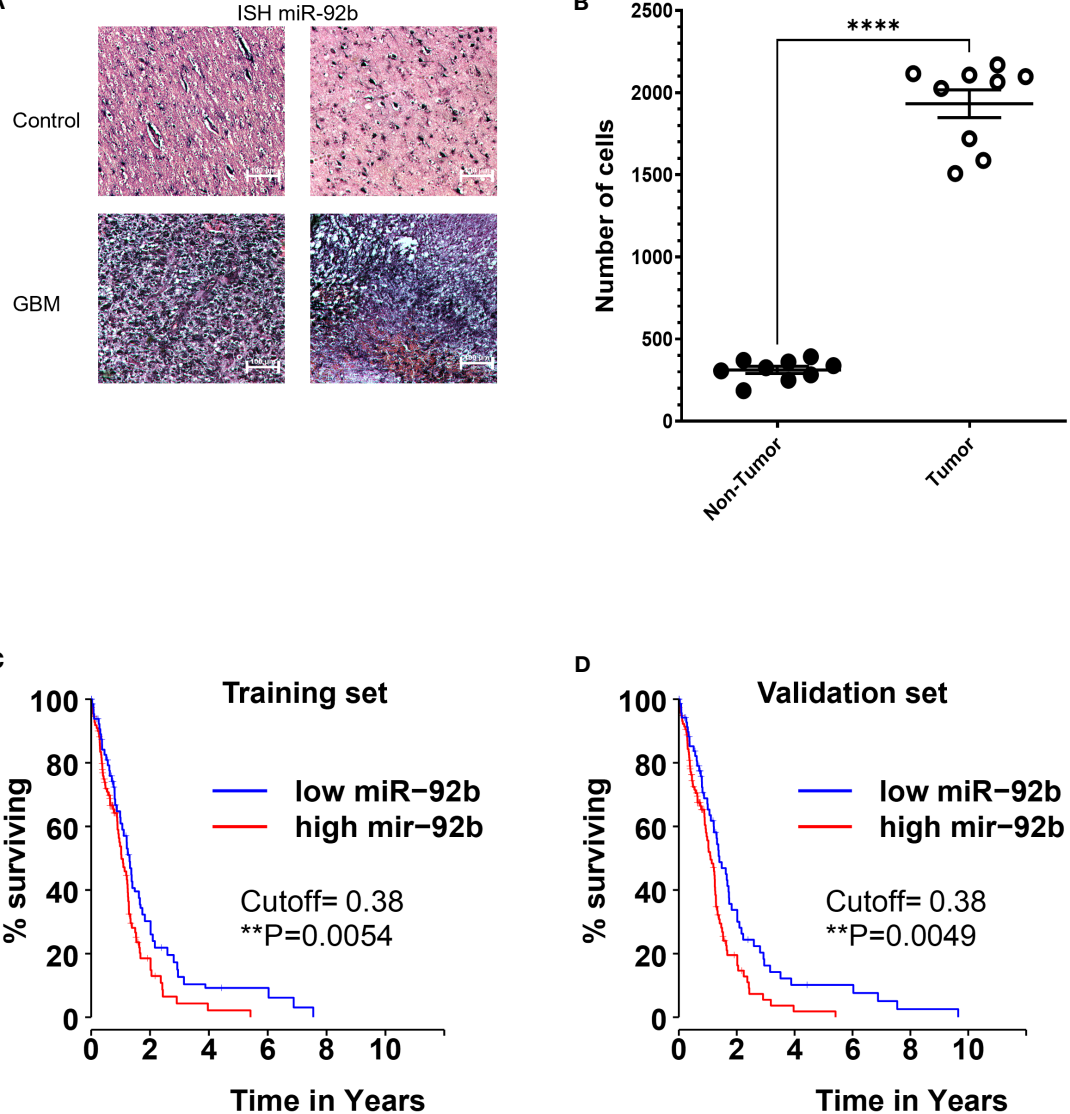
Expression of miR-92b in patient samples. **(A)** *In situ* hybridization (ISH) showing the miR-92b staining in two GBM and two control paraffin tissue samples. **(B)** graph showing the number of miR-92b-positive staining cells in control and GBM tissue samples. **(C, D)** Kaplan-Meier plot showing the correlation between the OS of glioma patients and the miR-92b mRNA levels. Data was extracted from the TCGA patient database. Averages ± SEM are shown for three independent experiments (t-test: n=3, **P<0.01 and ****P<0.0001).

ISH technique enables the detection and localization of miR-92b in GBM tumors, while retaining the tumor’s pathohistological features (**Figure 1A**). ISH staining showed that miR-92b expression levels were six times higher in GBM tissues when compared to normal brain tissues (****p<0.0001) (**Figure 1B**). Interrogation of the TCGA database showed that the overall survival (OS) of glioma patients with higher miR-92b levels live less compared with glioma patients with lower miR-92b levels (training set, p=0.0054) (**Figure 1C**). Statistical analysis with the validation set cohort (**Figure 1D**) corroborated these findings (p=0.0049).

### MiR-92b-OMIs reduced cell growth and proliferation in GBM cells

**Figure 2:**
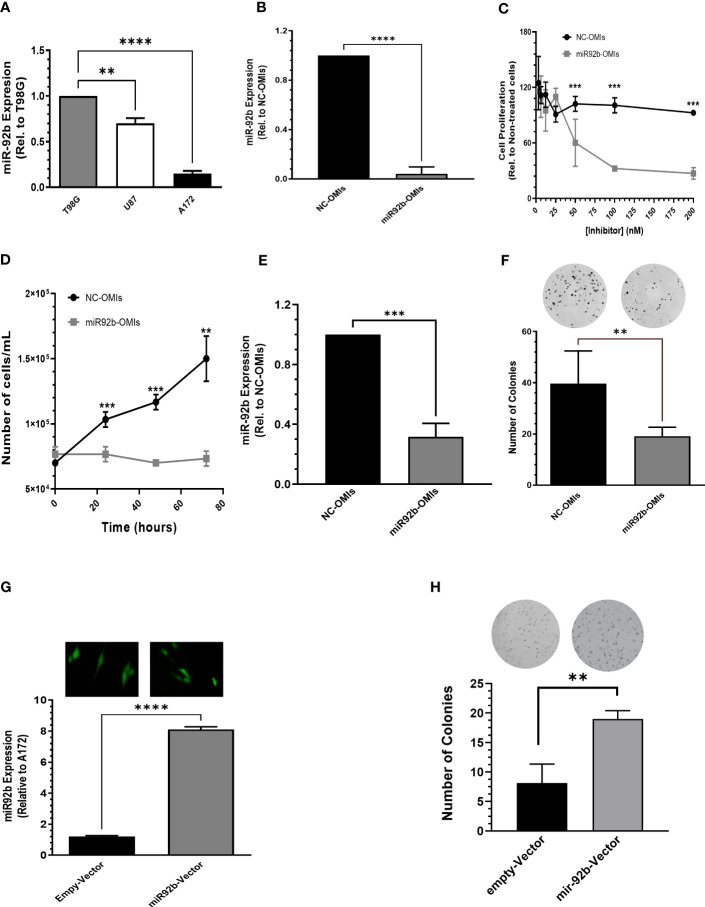
Effect of miR-92b deregulation on cell growth and proliferation. **(A)** MiR-92b expression in three human GBM cell lines (T98G, U87, and A172). Values were relative to the miR-92b expression in T98G cell line. **(B)** RT-PCR to confirm the decreased miR-92b expression after transient transfection of U87 cells with the miR-92b inhibitor (miR92b-OMIs) or a negative control inhibitor (NC-OMIs). **(C, D)** Dose-dependent and time-dependent assays after transient transfection of U87 cells with the miR-92b inhibitor (miR92b-OMIs) or a negative control inhibitor (NC-OMIs). **(E)** RT-PCR to confirm the decreased miR-92b expression after transient transfection of T98G cells with the miR-92b inhibitor (miR92b-OMIs) as compared with negative control inhibitor (NC-OMIs). **(F)** Transient transfection of miR-92b-OMI in T98G significantly reduced the number of colonies as compared with the NC-OMI. **(G)** stable transfection of a miR-92b in A172 cells with GFP-containing vectors (Green). The RT-PCR shows the expression of miR-92b in A172 cells transfected with the miR-92b-containing vector and with an empty-vector (EV). **(H)** The number of colonies formed with the miR-92b overexpressing cells were significantly higher as compared with EV-expressing cells. Averages ± SEM are shown for three independent experiments (t-test: n=3, **P<0.01, ***P<0.001 and ****P<0.0001).

Next, we studied the biological effects of targeting miR-92b in GBM cells. First, we compared miR-92b expression in three well-known human GBM cell lines (T98G, U87, and A172). The T98G cell line exhibited the highest expression of miR-92b, followed by U87 (25% lower than T98G, *P<0.05) and A172 (80% lower than T98G. ***P<0.001) (**Figure 2A**). U87 cells are tumorigenic in nude mice. Transient transfection of miR92b-OMIs in U87 cells reduced the MiR-92b levels by around ~95% (***P<0.001, **Figure 2B**) compared with the NC-OMIs. A dose-response experiment showed that transient transfection of U87 cells with 100 nM or 200 nM of miR92b-OMIs (final concentrations) significantly decreased cell viability to 67% and 48% (*P<0.05), respectively, when compared with the NC-OMIs (**Figure 2C**). Furthermore, we observed similar reductions in the cell number of U87 transfected cells with 100 nM of miR92b-OMIs after 24 hours (***p<0.001), 48 hours (**p<0.01), and 72 hours (***p<0.001) of drug incubation, as compared with the NC-OMI (**Figure 2D**).

Since colonies are difficult to observe with the U87 cells, we performed clonogenic assays with T98G cells. **Figure 2E** shows the knockdown efficiency of miR92b-OMIs transfection in this cell line compared to NC-OMIs (***p<0.001). Transfection of T98G cells with the miR92b-OMIs reduced the number of colonies to 20% (**p<0.01) compared to the NC-OMIs (**Figure 2F**). To confirm the role of miR-92b in cell growth and proliferation, we stable transfected a vector containing the miR-92b sequence in A172 cells. The transfection efficiency was observed by fluorescence microscopy images (GFP, green) taken at 20X magnification (**Figure 2G**). The increased miR-92b levels were assessed by real-time PCR. MiR-92b expression (miR-92b-3p) increased 8 times compared with EV clones (***P<0.001) (**Figure 2G**). MiR-92b overexpression increased the number of colonies by more than 50% (**p<0.01) as compared with EV clones (**Figure 2H**). Together, these results suggest that increased levels of miR-92b promote the cell growth and proliferation of GBM cells.

### MiR-92b-OMIs reduced cell migration and increased apoptosis of GBM cells

**Figure 3:**
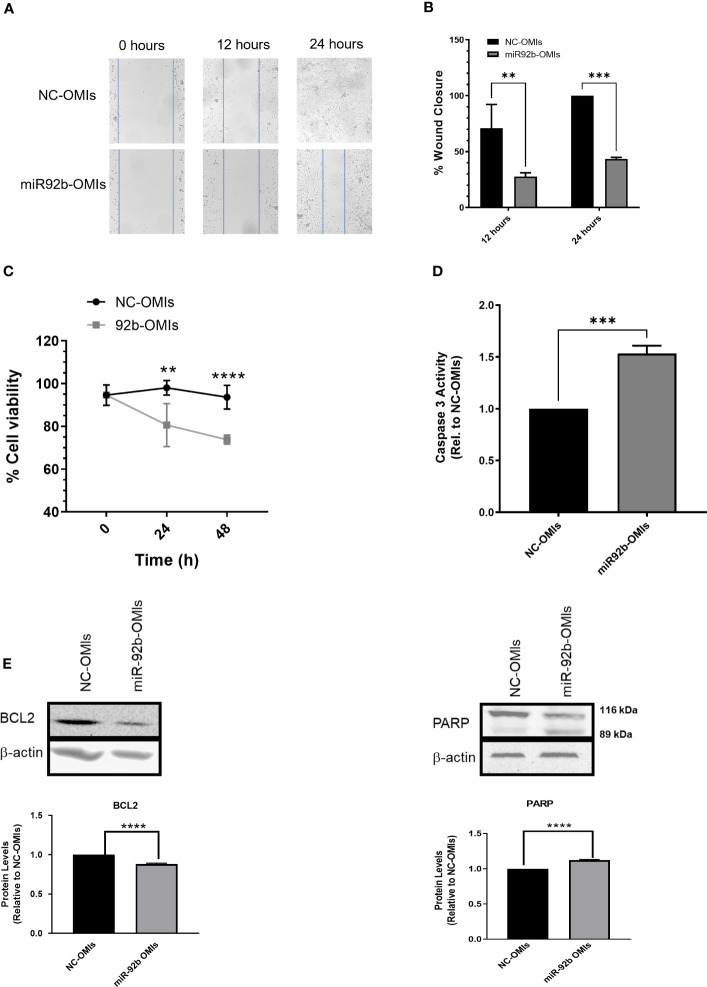
Effect of miR-92b knockdown in cell migration and apoptosis. **(A)** Migration assay in U87 cells transfected with miR-92b-OMIs or NC-OMIs at different time points (0h, 12h, and 24h). **(B)** Statistical analysis of the migration assay using U87 cells showing the wound closure percentage at different time points (12h & 24h) relative to NC-OMIs. **(C)** Trypan-blue exclusion assay in U87 cells transfected with miR-92b-OMIs or NC-OMIs (two-way ANOVA with n=3, **P<0.01, and ****p<0.0001). **(D)** Caspase-3 activity in U87 cells transfected with miR-92b-OMIs or NC-OMIs. **(E)** Western blots and densitometric analysis of the band’s intensity. Western blots were performed with 50 µg of protein extracted from U87 cells transfected with a NC-OMIs or the miR-92b-OMI. Averages ± SEM are shown for three independent experiments (t-test: n=3, **P<0.01, ***P<0.001, and ****P<0.0001).

Next, we assessed the effect of inhibiting miR-92b in cell migration using the Wound Healing Assay. Transient transfection of miR92b-OMIs in U87 cells showed lower wound closure rates than cells transfected with NC-OMIs (**Figures 3A, B**). Cells transfected with 50 nM of miR92b-OMIs closed about 50% of the wound gap compared to their NC-OMIs transfected cells (**Figures 3A, B**).

To assess the impact of miR92b inhibition on cell death, we counted the number of viable cells by the Trypan Blue exclusion assay. The number of alive cells was significantly reduced after 24-hr and 48-hr transfection of U87 cells with miR-92b OMIs as compared with NC-OMIs (**Figure 3C**). To determine the mechanism of cell death we performed a Caspase-3 Activity Assay in U87 cells transfected with OMIs. This experiment revealed that the miR92b-OMIs increased in approximately 50% (***p<0.001) the levels of DEVD-AFC cleavage induced by caspase-3 as compared with NC-OMIs transfected cells (**Figure 3D**). The increased levels of caspase-3 were accompanied by the reduction of the antiapoptotic protein BCl-2, and the typical cleavage of the 116-kDa poly-(ADP-ribose) polymerase PARP-1 into the 89 kDa and 24 kDa fragments (due to the small size, the fragment of 24-kDa is not observed in the membrane) (**Figure 3E**). These results suggest that targeting miR-92b has the potential to decrease cell migration and promote apoptosis in GBM cells.

### Prediction and validation of potential miR-92b target genes in GBM cells

**Figure 4:**
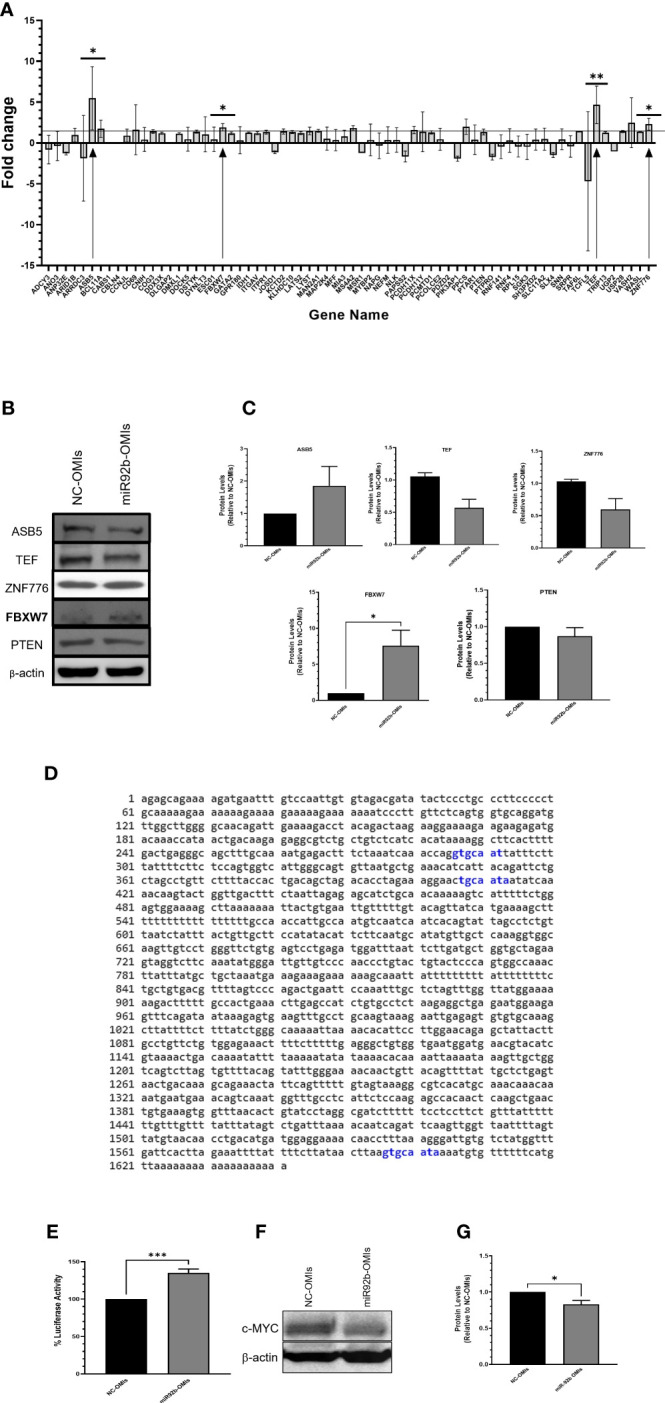
Identification of miR-92b downstream effectors. **(A)** U87 cells were transiently transfected with NC-OMIs or miR-92b-OMIs. RNA was isolated, cDNA was synthetized, and used for a 384-pre-disigned PCR plate with primers of the 70 (including PTEN) potential miR-92b-target genes. The arrows show the four genes exhibiting a fold change higher than 1.5. **(B)** western blots was performed with 50 µg of protein extracted from U87 cells transfected with a NC-OMIs or the miR-92b-OMI. β-actin was used as the loading control. **(C)** Quantification of the band intensities of the western blots showed in **(B)**. **(D)** Predicted binding sites of miR-92b 3p in the 3’ UTR of FBXW7 using the miRDB database. **(E)** Luciferase activity assay after transfecting U87 cells with the 3’UTR-containing vector of FBXW7 and OMIs (NC-OMI or miR-92b-OMI). Average ± SEM are shown for five independent experiments (n=5 and ***P<0.001). **(F)** Western blotting was performed as in B with a c-MYC antibody. **(G)** Quantification of the western blot intensity bands of c-MYC (n=3 and *P<0.05) by a t-test.

We performed an *in-silico* analysis using four different free internet-available miRNA prediction software to identify new target genes of miR-92b. First, we selected the top 250 genes in each of the four software. Then, we obtained 69 genes predicted by at least three of the software (Venn diagram **Supplementary Figure 1**). To validate these potential target genes, we isolated the total RNA of U87 cells transfected with the NC-OMIs or miR92b-OMIs and performed RT-qPCR in a 384-well plate, previously customized with the predicted miR-92b target genes. The expression changes caused by miR-92b inhibition (miR-92b-OMIs) in U87 cells were compared to the control cells, transfected with NC-OMIs (**Figure 4A**). For further validation, we selected genes with a fold change higher than 1.5. Only 4 out of the 69 genes increased the expression following miR-92b knockdown (**Figure 4A**). The changes in the mRNA expression of the four genes were statistically significant in all cases (*p<0.05). These genes included ASB5, TEF, ZNF776, and FBXW7 (**Table 1**). The changes in the mRNA levels of PTEN and NLK were lower than 1.5 and were not statistically significant (**Figure 4A**). These genes have been previously reported as miR-92b target in GBM cells (28, 29). Nevertheless, according to the four software and the miRTarBase database, FBXW7 was the only top predicted miR-92b target (**Supplementary Table 1**). Then, we measured the protein levels of the four genes by western blot analysis in NC-OMIs and miR-92b-OMIs U87 transfected cells. We used an antibody against PTEN. Only, the FBXW7 protein levels significantly increased (*p<0.05) when U87 cells were transfected with miR92b-OMIs (**Figures 4B, C**). The miRDB miRNA target prediction software showed three potential binding sites of miR-92b-3p to the 3’UTR region of FBXW7 (**Figure 4D**). We performed luciferase reporter assays to confirm that miR-92b binds directly to the 3’UTR of the FBXW7 mRNA. We observed that miR-92b-OMIs transfection led to a 35% (***p<0.001) increase in the relative luciferase activity compared to the NC-OMIs (**Figure 4E**). These results suggest that FBXW7 is a miR-92b target gene in U87 GBM cells. The FBXW7 is known to be involved in the ubiquitin-proteasome pathway that mediates ubiquitin-dependent proteolysis of oncoproteins like cyclin E1, c-Jun, Notch, and c-Myc (30, 31). **Figures 4F, G** shows that the c-MYC protein levels were significantly reduced when U87 cells were transfected with miR92b-OMIs.

**Table 1:** Biological role of the four genes of increased mRNA expression following miR-92b knockdown.

Gene symbol	Protein name	Protein function	Cell location	Cancer
ASB5	Ankyrin repeat and SOCS box protein 5	Involved in the Ubi conjugation pathway for proteosome-mediated degradation. Part of the SOCS family, which suppresses cytokine signaling.	Plasma membrane	Upregulated
TEF	Thyrotroph embryonic factor	Part of the PAR-bZIP transcription factors. A TSHB promoter transactivator. Involved in embryonic development.	Nucleus	Downregulated
ZNF776	Zinc finger protein 776	A DNA-binding transcription factor. Regulates the transcription of RNA Polymerase II.	Nucleoplasm and cytosol	Upregulated
FBXW7	F-box/WD repeat-containing protein 7	Controls proteosome-mediated ubiquitination of oncoproteins. Involved in phosphorylation dependent ubiquitination	Nucleoplasm and vesicles	Downregulated

(Suppressors of cytokine signaling), PAR (proline and acidic amino acid rich), bZIP (basic leucine zipper), TSHB (thyroid stimulating hormone β).

**Figure 5:**
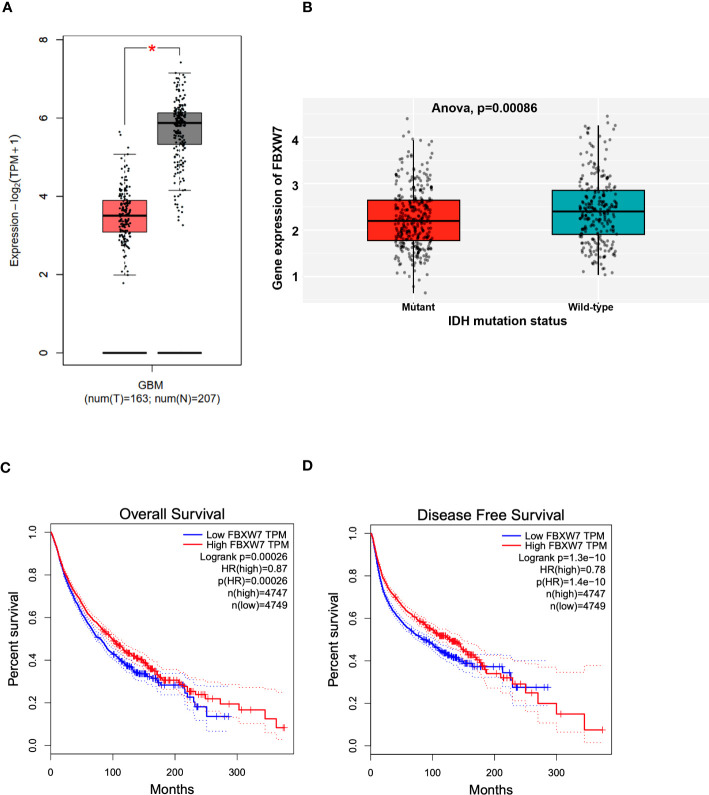
Clinical significance of FBXW7 in cancer. **(A)** Box plot showing the mRNA expression levels of FBXW7 in GBM patients and control samples. Figure generated with data available in the Gepia database. **(B)** Box plot showing the correlation between the FBXW7 mRNA expression levels and the IDH status in GBM patients. Figure generated with the GlioVis (CGGA) brain tumor patient database. **(C, D)** Kaplan-Meier plots showing the correlation between the FBXW7 mRNA levels and the overall survival (left) or disease-free survival (right). Survival curves generated with data available in the Gepia database.

To determine the potential clinical relevance of FBXW7, we interrogated the Gepia patient database (gepia2.cancer-pku.cn). The boxplot showed in **Figure 5A** shows that FBXW7 mRNA levels is lower in GBM patients as compared with normal controls (p<0.05). Interrogation of the Chinese Glioma Genome Atlas (CGGA; data set: mRNAseq_693) (CGGA is included in GlioVis) showed that GBM patients with isocitrate dehydrogenase (IDH) mutations express lower FBXW7 mRNA levels as compared with GBM patients with wild-type (WT) IDH (***p=0.00086) (**Figure 5B**). The CGGA includes molecular and clinical database of 625 low grade gliomas and 388 GBM patients. GBM patients with IDH mutations have a better outcome compared to those with WT IDH. The Rembrandt database (this database includes 28 non-tumor samples, 225 low grade gliomas and 219 GBMs) showed that FBXW7 is significantly higher in non-tumor samples as compared with oligodendrogliomas, astrocytomas, and GBM patients (**Supplementary Figure 2A**). The Kaplan-Meier plot obtained in the Gepia patient database shows that the overall survial (OS; p=0.00026) (**Figure 5C**) and the progression-free survival (PFS; p=1.3E-10) (**Figure 5D**) are higher for GBM patients with higher FBXW7 as compared with GBM patients with lower FBXW7 levels. The Rembrandt database confirmed these findings (**Supplementary Figure 2B**).

### Liposomal miR-92b-OMIs reduced *in vivo* tumor growth

**Figure 6:**
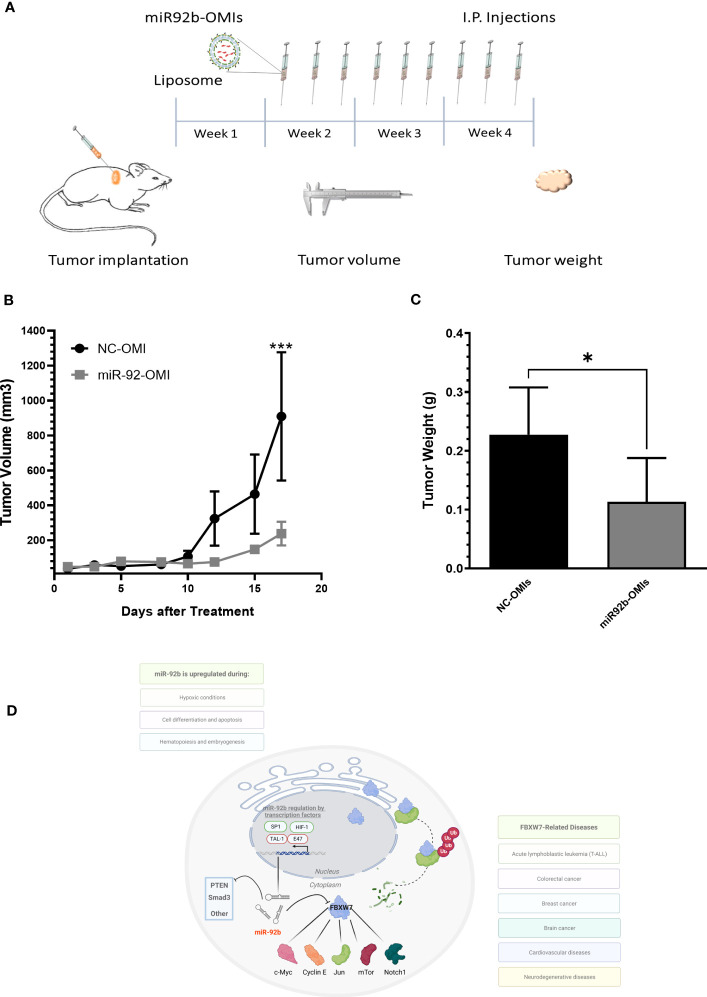
*In vivo* therapeutic efficiency of a liposomal miR-92b-OMI formulation. **(A)** Timeline diagram of the experimental design. **(B)** One-week after cell implantation, mice were intraperitoneally injected three times a week with liposomes containing miR-92b-OMIs (2.5 µg/per dose) or NC-OMI (2.5 µg per dose). **(C)** Tumor volumes were calculated measuring tumor dimensions. **(C)** At the end of the experiment, mice were euthanized, and tumors were extracted and weighted. Averages ± SEM are shown for NC-OMIs (N=5), and miR92b-OMIs (N=7) treated mice (*P<0.05 and ***P<0.001) by a t-test. **(D)** Diagram showing the miR-92b downstream effectors in GBM cells.

To determine the therapeutic consequences of miR-92b-OMIs, we treated GBM-bearing mice with liposomes containing either NC-OMIs or miR-92b-OMIs. The liposomal formulation used in this study has already been described elsewhere (20, 24, 26). One week before treatment, human U87 cells were implanted into the dorsal flank of nude mice to develop a subcutaneous GBM Xenograft mouse model. GBM-bearing mice were divided into NC-OMIs (N=5) and miR-92b-OMIs (N=7). Three times a week, intraperitoneal (i.p.) injections of liposomes (containing 2.5 µg of NC-OMIs or 2.5 µg of miR92b-OMIs) were administered. Tumor volumes were measured in each treatment day (measuring the dimensions with a caliper), and tumor weights were assessed at the end of the experiment (**Figure 6A**). Treatment of GBM-bearing mice with miR92b-OMIs decreased tumor growth compared to their control groups (**Figure 6B**). Specifically, treatment with miR92b-OMIs for seventeen days significantly reduced tumor volume by 78% (**P<0.01) compared to treatment with NC-OMIs (**Figure 6B**). At the end of the experiment a significant tumor weight reduction of 50% was observed in liposomal miR92b-OMIs treated mice compared to Liposomal NC-OMIs treatment (*P<0.05) (**Figure 6C**).

## Discussion

The major finding of this study is that miR-92b regulates FBXW7, a previous well described tumor suppressor gene in multiple types of cancers such as acute lymphoblastic leukemia (T-ALL), colorectal cancer, breast cancer, and brain cancer (32, 33). We also showed that targeting miR-92b with an OMI-liposomal formulation reduced tumor growth in a subcutaneous GBM mouse model. Early reports of Wu et al. showed that miR-92b expression was higher in GBM patient samples compared with control brains (22). In the present study, using *in situ* hybridization (ISH) we obtained similar results as the miR-92b staining was significantly higher as compared with normal brain tissues. Furthermore, interrogation of the TCGA database confirmed that GBM patients with higher miR-92b have reduced prognosis compared with GBM patients with lower miR-92b expression levels.

An early miRNA expression profile showed that miR-92b was among the top thirteen dysregulated miRNAs and it was higher in all astrocytoma grades as compared with normal tumor samples (20). Aberrantly increased levels of miR-92b have also been reported in breast and bladder cancers, and melanoma-derived extracellular vesicles (33–35). Moreover, Wu et al., Xu et al., Li et al., and Wang et al. published that knockdown of miR-92b inhibited cell growth and proliferation, and induced apoptosis and cell cycle arrest in GBM cells (22, 28, 36, 37). Additionally, stable transfected GBM cells with a vector that knocked down miR-92b reduced the proliferation rate in a subcutaneous GBM mouse model (28). Based on this information miR-92b is considered an oncomiR. However, in pancreatic cancer, miR-92b acts as a tumor suppressor gene demonstrating possible tissue-specific effects (38). This preliminary information, together with our results indicates that miR-92b is a potential target not only for GBM but for other brain tumor types.

Our study showed that targeting miR-92b strongly reduced cell viability, cell proliferation, and cell migration, and induced apoptosis. The ectopic expression of miR-92b produced opposite results. Wu et al. showed that miR-92b knockdown also inhibited cell cycle progression in the G0/G1 phase by up-regulation of the TGF-beta/smad3/p21 signaling pathway (22). The reduced levels of CDK4 and cyclin D3 confirmed that targeting miR-92b promotes cell cycle arrest as markers of the G1 phase. Although the changes observed in cell proliferation and migration upon miR-92b knockdown were noticeable, the effect on apoptosis was lesser. These results suggest that knockdown of miR-92b could induce another mechanism of cell death. In fact, it has been reported that miR-92b promotes autophagy in breast cancer cells (39). This hypothesis should be further investigated in GBM cells.

Various miR-92b (3p) target genes have been reported (37, 40). Wu et al. showed that smad3 is a miR-92b target gene in U87 cells (22). In fact, in the same study, the smad3 levels were reduced in GBM samples (22). Xu et al. showed that in T98G and LN229 GBM cells PTEN is a direct target of miR-92b (28). Wang et al. reported that knockdown of miR-92b reduced cell growth and proliferation and induced apoptosis in GBM cells by targeting Nemo-like kinase (NLK) (29). Li et al. showed that Dickkopf-3 (DKK3) gene is a miR-92b target gene in U87 cells (36). More recently, Wang et al. showed that reduced expression of miR-92 promotes proliferation, migration, invasion and apoptosis in GBM cells by targeting neogenin (NEO1) (41). In addition, miR-92b transcription is regulated by transcription factors like TAL1-E47 during hematopoiesis and embryogenesis, SP1 during cell differentiation and apoptosis, and HiF1 during hypoxic conditions (42–44). In our study, we performed a rigorous bioinformatics analysis combined with real-time PCR, and western blots to identify potential miR-92b target genes. Real-time PCR and western blots showed that, following miR-92b knockdown the FBXW7 were increased at the mRNA and protein levels, respectively. However, we observed that the protein levels of PTEN did not change upon miR-92b targeting. In fact, the only top predicted miR-92b target of the four software challenged was FBXW7. Nevertheless, the number of passages of the U87 cells or the acquisition of gene mutations during cell culture could explain these differences. Moreover, the toxicity of transfection reagents may impact protein expression. Nevertheless, our luciferase reporter assays confirmed that FBXW7 is a direct target of miR-92b.

FBXW7, also known as the hCDC4, is a protein involved in the ubiquitin-proteasome pathway that mediates ubiquitin-dependent proteolysis of key oncoproteins including cyclin E1, c-Jun, Notch, and c-Myc (**Figure 6D**) (32, 34, 45). FBXW7 plays a vital role in cellular division, and its downregulation is linked to cancer progression (30). Moreover, the loss of FBXW7 has been extensively studied and consistently linked to the pathogenesis of cancer, as well as tumor metastasis, poor clinical outcomes, and resistance to a range of cancer treatments including chemotherapy, radiation therapy, and immunotherapy (45, 46). Studies by Cai et al. demonstrated that upregulation of FBXW7 reduces renal cancer metastasis and epithelial-mesenchymal transition *in vitro* (46). Similar results were found in colorectal cancer where FBXW7 decreased tumor burden of colorectal carcinoma by negative regulation of enolase-1 (ENO1), NK2, mTOR, and PTEN (47, 48). Therefore, a possibility is that the reduced PTEN protein levels following miR-92b overexpression reported by Xu et al. are mediated by FBXW7. The *in-silico* analysis predicted several potential miRNA target genes. Therefore, upregulation of miR-92b could exert its oncomiR effects by targeting other target genes. The molecular pathway leading to miR-92b overexpression should also be investigated.

Hagerdon et al. used qRT-PCR in RNA isolated from human glioma biopsies and found that the FBXW7 expression was significantly reduced in GBM tissues compared to normal healthy brain tissues (49). By using the GEPIA patient database we found that the FBXW7 mRNA levels were lower in GBM patients compared with normal brain samples. The finding of a correlation between FBXW7 mRNA expression and the IDH status suggests that FBXW7 might serve as a predictive indicator for drug response in GBM. We also found that the FBXW7 mRNA levels correlated with the OS and PFS in GBM patients. Together, this evidence confirms our results and indicates that FBXW7 is certainly a tumor suppressor gene and that its mRNA levels could be proposed as diagnostic and prognostic markers in various cancer types, including and specially in GBMs (32, 34, 45).

Our study also found that multiple *i.p*. injections of our liposomal-miR92b-OMIs formulation inhibited tumor growth in a subcutaneous GBM mouse model. Wang, et al. and Wu et al. locally injected a miR-92b OMIs in a subcutaneous GBM mouse model and observed a significant reduced tumor volume compared with a NC-OMI (22, 37). Our liposomal formulation (DOPC, cholesterol, DSPE-PEG-2000) to encapsulate miR92b-OMIs showed negligible toxicity both *in vitro* and *in vivo*; and no detectable immune responses (26). Also, these liposomes efficiently delivered c-MYC-targeted siRNA in a xenograft mouse model of ovarian cancer and miR-143-OMIs in a subcutaneous GBM mouse model (24, 26). Several studies have utilized mouse models to investigate the potential of targeting miR-92b for cancer therapy. Our study, however, has shown that liposomal-miR92b-OMIs formulation can effectively inhibit tumor growth in a subcutaneous GBM mouse model without causing any significant toxicity or immune responses. Future studies should use our liposomal-miR-92b-OMI formulation in orthotopic xenograft models of GBM for higher clinical relevancy.

## Conclusions

In summary, we observed that miR-92b is increased in GBM samples and its expression correlates well with the overall survival of GBM patients. Targeting miR-92b reduces cell growth and migration, induces apoptosis, and inhibits tumor growth in a subcutaneous GBM mouse model. Our study reveals that miR-92b regulates FBXW7 expression in GBM cells, and that the oncogenic-like effects of miR-92b may be partially mediated through suppression of FBXW7. FBXW7 is also clinically relevant, as lower mRNA levels of this gene are associated with poor GBM prognosis. The multifaceted role of miR-92b in regulating multiple cellular pathways makes it an attractive target for GBM therapy. These findings have provided important evidence to continue moving our liposomal miR-92b-OMI formulation into preclinical studies using patient derived xenograft (PDX) and orthotopic GBM mouse models.

## Data availability statement

The original contributions presented in the study are included in the article/**Supplementary Material**. Further inquiries can be directed to the corresponding author.

## Ethics statement

Ethical approval was not required for the studies on humans in accordance with the local legislation and institutional requirements because only commercially available established cell lines were used. The animal study was approved by Institutional Animal Care and Use Committee (IACUC). The study was conducted in accordance with the local legislation and institutional requirements.

## Author contributions

PV-M conceived and supervised the overall study and edited the final version of the manuscript. NG-R contributed to the experimental design, conducted the experiments, prepared the figures, and wrote part of the manuscript. AS-Á conducted experiments, wrote part of the manuscript, prepared and formatted figures. NG-R and AS-Á contributed equally. EL-D contributed to the experimental design, performed growth assays, contributed in the TCGA patient data analysis, and performed *in vivo* experiments. FV performed *in vivo* experiments. CR-V and FV performed *in situ* hybridization experiments. YS-R performed Western blot experiments and revised figures. RR performed cell growth and migration assays and interrogated the Gepia patient database. All authors were actively involved in the preparation of the manuscript with the supervision of PV-M. All authors contributed to the article and approved the submitted version.
