# Supplementary material for: MicroRNA-92b targets tumor suppressor gene FBXW7 in glioblastoma
Source: Front Oncol. 2023 Sep 11;13:1249649. doi: 10.3389/fonc.2023.1249649 (PMC10518455; doi:10.3389/fonc.2023.1249649)
Supplement: Supplementary file 1 [file Presentation_1.pptx]

## Slide 1
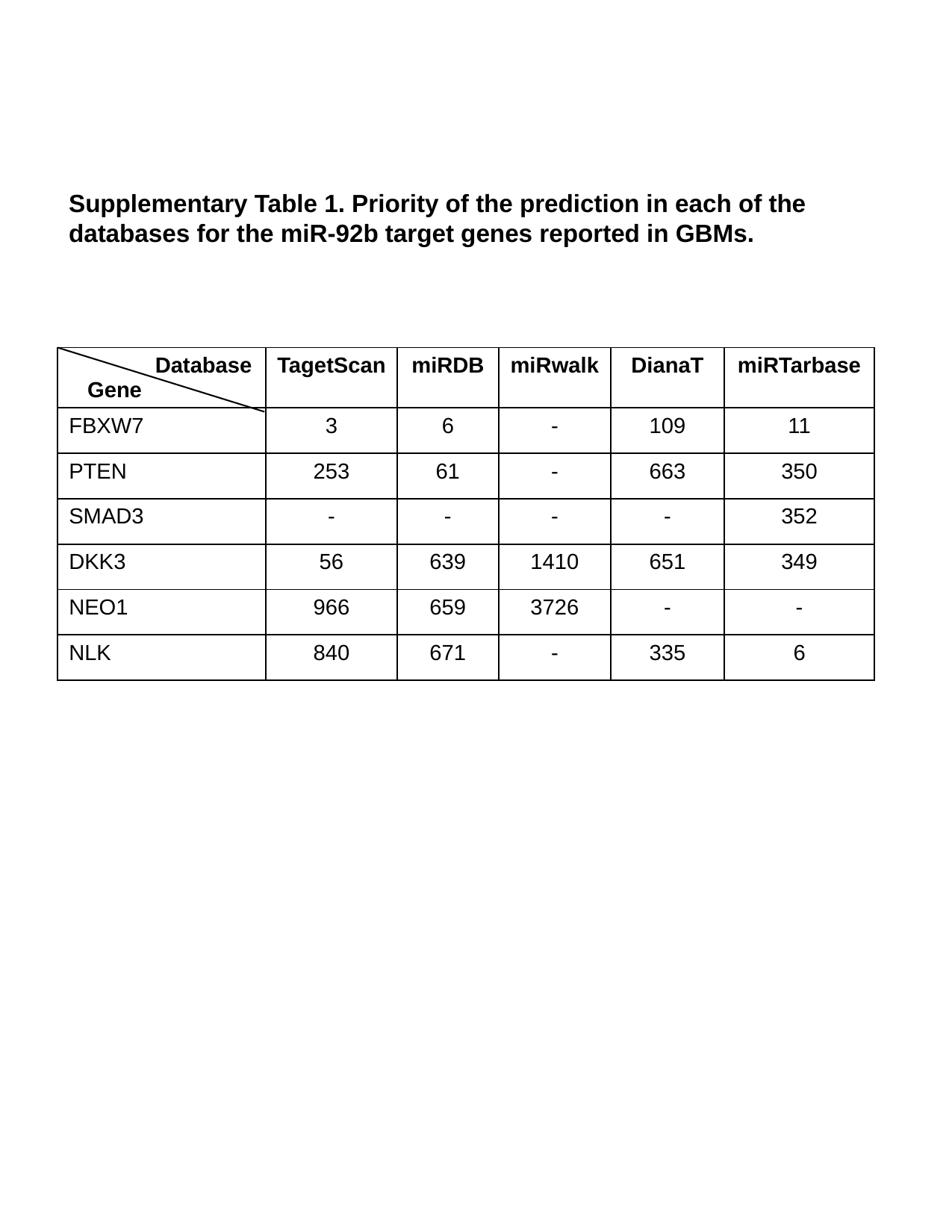

Supplementary Table 1. Priority of the prediction in each of the databases for the miR-92b target genes reported in GBMs.
| Database Gene | TagetScan | miRDB | miRwalk | DianaT | miRTarbase |
| --- | --- | --- | --- | --- | --- |
| FBXW7 | 3 | 6 | - | 109 | 11 |
| PTEN | 253 | 61 | - | 663 | 350 |
| SMAD3 | - | - | - | - | 352 |
| DKK3 | 56 | 639 | 1410 | 651 | 349 |
| NEO1 | 966 | 659 | 3726 | - | - |
| NLK | 840 | 671 | - | 335 | 6 |

## Slide 2
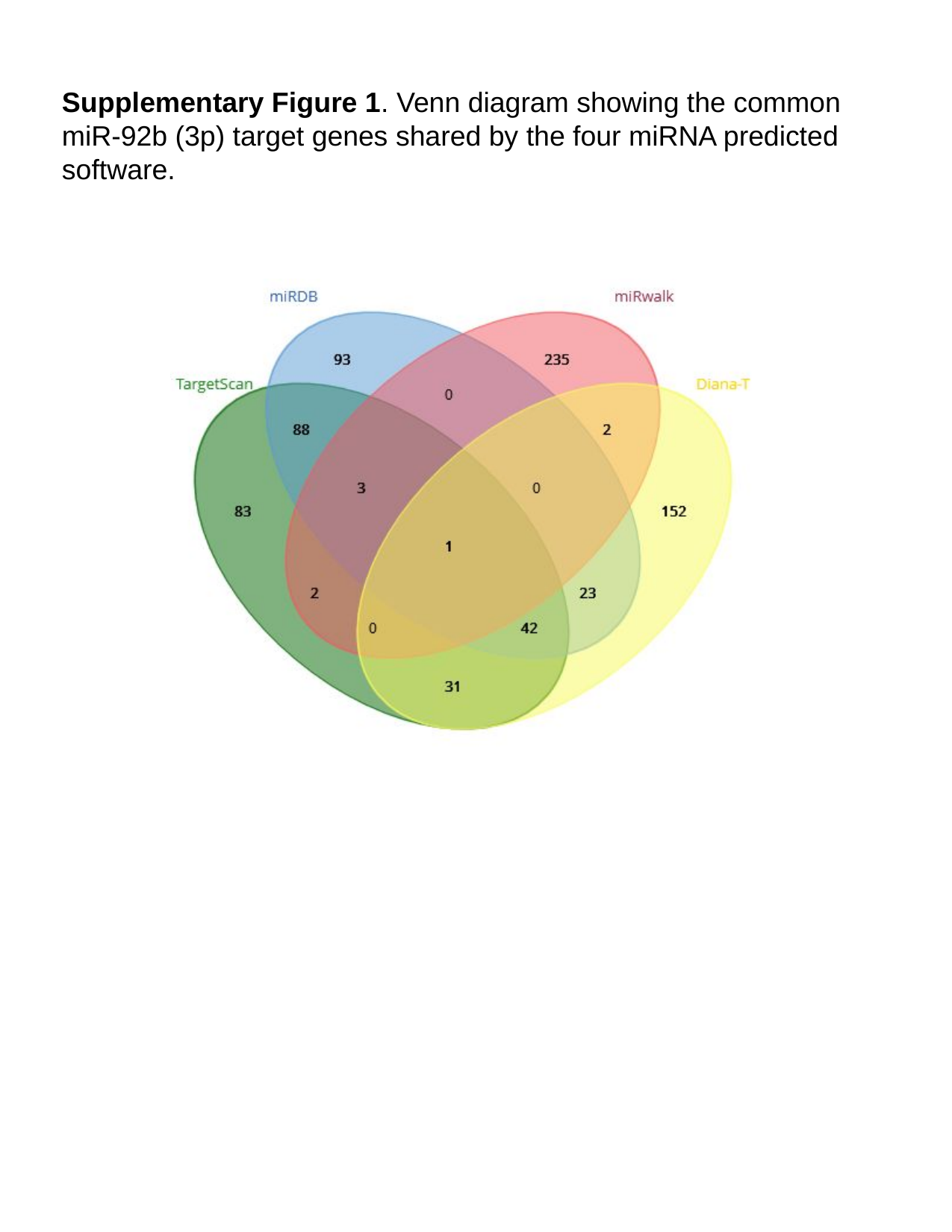

Supplementary Figure 1. Venn diagram showing the common miR-92b (3p) target genes shared by the four miRNA predicted software.

## Slide 3
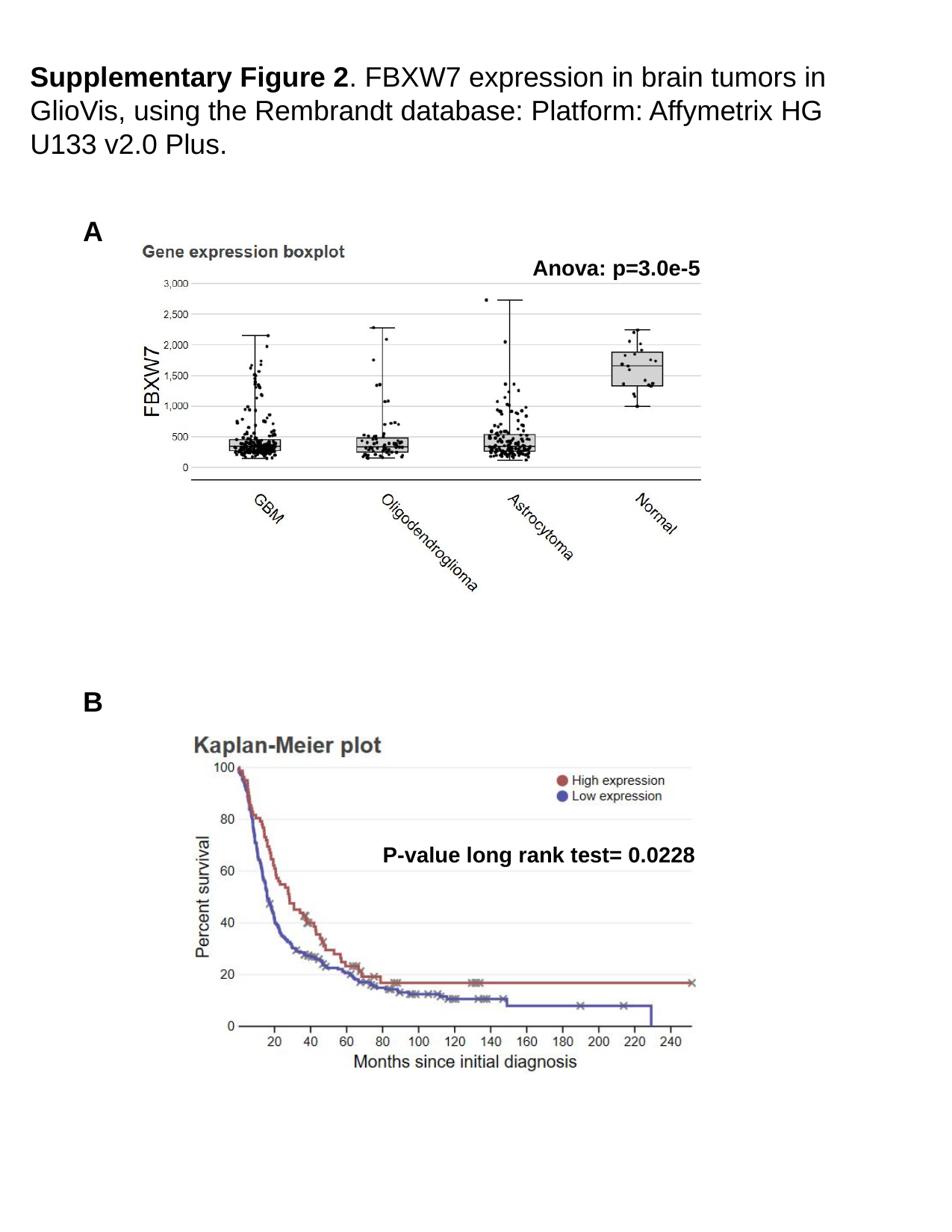

Supplementary Figure 2. FBXW7 expression in brain tumors in GlioVis, using the Rembrandt database: Platform: Affymetrix HG U133 v2.0 Plus.
A
B
Anova: p=3.0e-5
P-value long rank test= 0.0228
